# Comparative Efficacy of Horse and Chicken Serum for the In Vitro Cultivation of *Mycoplasma hyorhinis* Clinical Isolates

**DOI:** 10.3390/pathogens14101056

**Published:** 2025-10-19

**Authors:** Yi-Chia Li, Yu-Wei Tseng, Wei-Hao Lin, Chao-Nan Lin, Ming-Tang Chiou

**Affiliations:** 1 Department of Veterinary Medicine, College of Veterinary Medicine, National Pingtung University of Science and Technology, Pingtung 912301, Taiwan; yichavet@gmail.com (Y.-C.L.); yuwei7961@gmail.com (Y.-W.T.); whlin@mail.npust.edu.tw (W.-H.L.); cnlin6@mail.npust.edu.tw (C.-N.L.); 2 Animal Disease Diagnostic Center, College of Veterinary Medicine, National Pingtung University of Science and Technology, Pingtung 912301, Taiwan; 3 Sustainable Swine Research Center, National Pingtung University of Science and Technology, Pingtung 912301, Taiwan

**Keywords:** *Mycoplasma hyorhinis*, horse serum, chicken serum, culture medium optimization, CCU (color changing unit)

## Abstract

*Mycoplasma hyorhinis* is an important respiratory pathogen in swine, yet optimal culture conditions for high-yield propagation remain undefined. This study compared horse serum (HS) and chicken serum (CS) at graded concentrations (10%, 20%, 30%) for their ability to support in vitro growth of four clinical *M. hyorhinis* isolates (strains A, B, C, and D). Cultures were prepared in modified Friis medium, and growth performance was assessed by final titer (color changing unit, CCU/mL) and time-to-detection at 10^2^ and 10^4^ CCU/mL. All media supported growth, but HS consistently outperformed CS in both yield and growth kinetics. The highest titers (10^9^ CCU/mL) and shortest detection times (3.6–6 days) were observed in 20% HS for most strains. Increasing HS concentration to 30% reduced yield for several strains, suggesting a concentration-dependent inhibitory effect. CS demonstrated limited but strain-dependent growth support, with comparable performance to HS for strain B at lower thresholds. These findings identify 20% HS as an optimal supplement for efficient *M. hyorhinis* cultivation, while highlighting the potential of CS as a cost-effective alternative under certain conditions, with implications for diagnostic reagent production and vaccine development.

## 1. Introduction

Among the mycoplasmal species that most severely impact swine production are *Mycoplasma hyopneumoniae*, *Mycoplasma hyosynoviae*, and *Mycoplasma hyorhinis* [1], which are the causative agents of swine enzootic pneumonia (SEP), arthritis/synovitis, and porcine polyserositis, respectively. Infections with these pathogens often result in growth retardation, predispose to secondary bacterial infections, and exacerbate diseases when co-infecting with other pathogens, leading to substantial economic losses for the global swine industry [2,3]. While respiratory disease in pigs has traditionally been associated with *M. hyopneumoniae*, recent cases of enzootic pneumonia-like lesions caused solely by *M. hyorhinis* have emerged in Taiwan [4], and herd-level prevalence exceeding 30% has been reported in mainland China [5], indicating the escalating importance of *M. hyorhinis* in swine respiratory disease.

Mycoplasmas are known as the smallest self-replicating prokaryotes. They are characterized by the lack of a cell wall and are enclosed by a single cell membrane composed of phospholipids and proteins, surrounding a double-stranded DNA genome [6]. Their genomes are notably small, and this genetic simplicity corresponds to a reduced metabolic capacity, making mycoplasmas heavily reliant on the host or exogenous sources for essential nutrients, particularly cholesterol and fatty acids [7,8]. Consequently, many *Mycoplasma* species are fastidious organisms characterized by slow growth and complex nutritional requirements that together make in vitro cultivation challenging [8,9,10]. This cultivation challenge not only impedes high-titer yield required for antigen production and vaccine development but also slows clinical diagnostics and routine laboratory isolation, for which rapid growth kinetics are particularly important.

The development of the liquid Friis medium in the 1970s represented a milestone for *mycoplasma* cultivation, containing components such as yeast extract and animal serum [11]. Horse serum has been traditionally employed as a key supplement to fulfill the nutritional demands of mycoplasmas. However, its use is associated with two major drawbacks: high cost and significant batch-to-batch variability in growth-supporting ability, both of which lead to inconsistent experimental outcomes [12]. Over the years, studies have explored alternative supplements to optimize growth media for various mycoplasmas. For instance, horse serum was identified as a critical factor for biomass production of *M. gallisepticum* [13], while the growth-promoting activity of commercial bovine serum fraction (BSF) for *M. pneumoniae* was correlated with its cholesterol content [14]. In a study optimizing culture media for *M. hyopneumoniae*, porcine serum was found to significantly affect protein production [15]. Furthermore, egg yolk extract has been demonstrated as a stable, inhibitor-free, and promising substitute for horse serum in the cultivation of *M. pneumoniae* [16]. In the search for a practical alternative, chicken serum (CS) presents a compelling option, given its potentially lower cost and broader availability in some regions. To date, no comparison of chicken versus horse serum at graded concentrations has been performed for *M. hyorhinis*, leaving the optimal supplement undefined. Therefore, the present study was designed to systematically evaluate and compare the efficacy of different concentrations of horse serum versus chicken serum in supporting the in vitro growth of *M. hyorhinis*.

## 2. Materials and Methods

### 2.1. Mycoplasma hyorhinis Strain Isolation and Identification

Four clinical isolates of *M. hyorhinis*, designated as strains A (D109198_RLC), B (D109602_RLC), C (D110881_RLC), and D (D110216_LLC), were utilized in this study. These strains were originally isolated from SEP-like lung lesions of field pigs at the Animal Disease Diagnostic Center of National Pingtung University of Science and Technology. Strains A and B were isolated in 2020, while strains C and D were isolated in 2021. Following isolation and purification, identification was conducted. Nucleic acid including DNA and RNA (total nucleic acid) was extracted from 200 µL of culture using the MagNA Pure 96 DNA and Viral NA Small Volume Kit on an automated MagNA Pure 96 Instrument (Roche, Mannheim, Germany), according to the manufacturer’s protocol. The nucleic acid was eluted in 50 µL of elution buffer. The identity of *M. hyorhinis* was then confirmed by a probe-based quantitative PCR (qPCR) assay on a LightCycler^®^ 96 Instrument (Roche, Basel, Switzerland). The assay utilized the forward primer Mhr-97F (5′-CCAAGACGATGATGTTTAGCC-3′), the reverse primer Mhr-97R (5′-AAATTCCTTACTGCTGCCTC-3′), and the Universal ProbeLibrary Probe #118 (Roche, Basel, Switzerland); sequence: 5′-CACTGGGA-3′) [17]. Confirmed isolates were stored as frozen stocks in a −80 °C freezer until further use.

### 2.2. Culture Revival and Working Stock Preparation

For subsequent experiments, cryopreserved stocks were revived using a proprietary formulation of modified Friis medium, developed and kindly provided by the Agricultural Technology Research Institute (Hsinchu, Taiwan); its detailed composition is not disclosed due to commercial confidentiality. Briefly, a 300 µL aliquot of a thawed stock was inoculated into 3 mL of the medium and incubated aerobically at 37 °C until the color changed from red to yellow. The identity of the revived culture was re-confirmed as *M. hyorhinis* using the qPCR assay described previously. The revived culture was then subcultured once to generate a high-titer working stock for the experiments.

### 2.3. Preparation of Experimental Culture Media

A serum-free basal medium was prepared based on the Friis medium formulation [11], with modifications. Briefly, the basal medium was composed of a laboratory-prepared basal broth (60% *v*/*v*) based on the Friis formulation [11], yeast extract solution (20% *v*/*v*, Sigma-Aldrich, St. Louis, MO, USA), and deionized water, and was sterilized by autoclaving. After the mixture cooled to room temperature, a 100× supplement solution was aseptically added to a final concentration of 1×. This 100× supplement was prepared in the laboratory and consisted of D-glucose (50 g/L, Sigma-Aldrich, St. Louis, MO, USA) and L-Arginine HCl (10 g/L, Sigma-Aldrich, St. Louis, MO, USA) dissolved in deionized water. This basal medium was then supplemented with either chicken serum (CS) (Thermo Fisher Scientific, Waltham, MA, USA) or horse serum (HS) (Cytiva, Marlborough, MA, USA) to final concentrations of 10%, 20%, or 30% (*v*/*v*) according to six distinct media formulations.

### 2.4. Determination of Working Stock Concentration by Color Changing Unit (CCU) Assay

The concentration of viable mycoplasmas in each of the four final working stocks was determined independently using a terminal dilution, color changing unit (CCU) assay. Each stock was serially ten-fold diluted, and aliquots were incubated in triplicate aerobically at 37 °C. The CCU titer (CCU/mL) for each working stock was calculated as the reciprocal of the highest dilution that exhibited a distinct color change after 30 days. The resulting titers for the working stocks of Strain A, B, C, and D were 3.67 × 10^6^, 3 × 10^9^, 3 × 10^8^, and 3 × 10^4^ CCU/mL, respectively.

### 2.5. Comparative Analysis of Media Performance by Time-to-Detection Assay

The entire comparative analysis was performed independently for each of the four *M. hyorhinis* strains. For each strain, a time-to-detection assay was employed to compare the performance of the six media formulations in three replicates. For each replicate, a set of test cultures was prepared corresponding to a ten-fold serial dilution of the respective working stock. This was achieved by inoculating 2.7 mL of a specific medium with 300 µL of the appropriate working stock dilution. All cultures were incubated aerobically at 37 °C and inspected daily for color change up to 30 days. For each medium formulation, an uninoculated tube was concurrently incubated as a negative control. These controls consistently exhibited no color change throughout the 30-day incubation. Two parameters were recorded: (i) Final Growth Yield: Determined by the endpoint titer (the highest dilution showing a positive color change) for each medium at the end of the observation period. (ii) Growth Kinetics: Assessed by ‘time-to-detection’, defined as the number of incubation days required for the culture titer to first reach or exceed two predetermined thresholds: 10^2^ and 10^4^ CCU/mL.

### 2.6. Statistical Analysis

All assays for each strain-medium combination were performed in three independent biological replicates. All raw data were log-transformed prior to calculation and are presented as the mean ± standard deviation (SD) of the transformed data. A normality test was performed prior to statistical analysis, and as the data were not normally distributed, a non-parametric approach was chosen. The Kruskal–Wallis test was applied to compare final endpoint titers and ‘time-to-detection’ among the six media groups for each strain. For post hoc analysis, the uncorrected Dunn’s test was chosen due to frequent identical values within triplicates. All analyses were performed using GraphPad Prism version 10.5 (GraphPad Software, Boston, MA, USA), and a *p*-value < 0.05 was considered statistically significant.

## 3. Results

The growth performance of the four *M. hyorhinis* isolates in media supplemented with either horse serum (HS) or chicken serum (CS) at three different concentrations was evaluated based on final growth yield and growth kinetics. A comprehensive summary of these results, including final titers and time-to-detection values, is presented in Table 1.

### 3.1. Final Growth Yield

To compare the efficacy of different sera for the cultivation of *M. hyorhinis*, the final growth yield of four clinical isolates (Strains A, B, C, and D) was determined. All groups successfully supported the growth of all four *Mycoplasma* strains, yielding final titers that ranged from approximately 10^2^ to 10^9^ CCU/mL. Notably, the maximum titers (10^9^ CCU/mL) were achieved by Strain A in media supplemented with 20% and 30% HS, and by Strain B with 20% HS. Conversely, the lowest titers (10^2^ CCU/mL) were observed in Strain C with 30% HS and 30% CS, and in Strain D with 30% HS and all CS-supplemented media. Among the strains, Strain A generally exhibited the highest or equal final titers across all conditions, whereas Strain D consistently yielded the lowest or equal titers. Furthermore, for Strains A, B, and D, growth in HS-supplemented media resulted in titers that met or exceeded the initial titers of the working stocks, while growth in all CS-supplemented media produced titers below the initial working stocks titers for all four strains.

The impact of serum concentration varied between serum types and among the strains. In HS-supplemented media, increasing the concentration from 10% to 20% resulted in a substantial 100- to 1000-fold increase in titers for Strains A and B; this effect was not observed for Strains C and D. However, a further increase to 30% HS led to a notable decrease in titers (100- to 1000-fold) for Strains B, C, and D compared to the 20% HS condition. In contrast, for media supplemented with CS, the final titers for Strains A and D did not exhibit significant variation with changes in serum concentration. For Strain C, however, the titer in 30% CS was approximately 100-fold lower than that in 20% CS.

**Figure 1:**
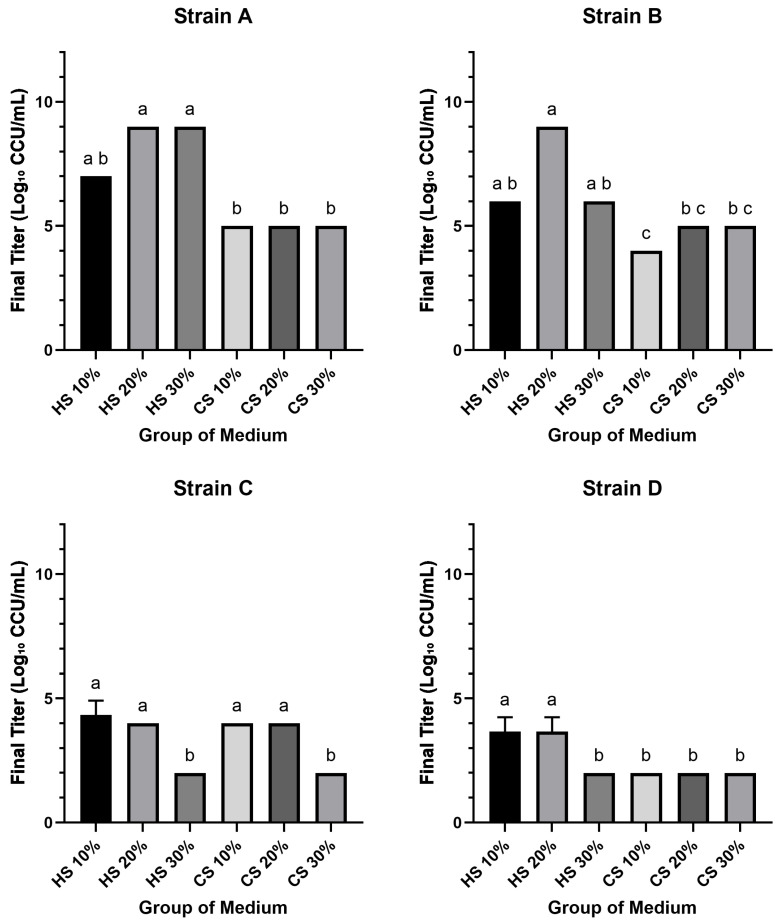
Final growth yield of four *M. hyorhinis* isolates in media supplemented with different sera. The final titer of four clinical isolates was determined after 30 days of incubation in modified Friis medium supplemented with 10%, 20%, or 30% of either horse serum (HS) or chicken serum (CS). The four panels show the results for Strains A, B, C, and D. Bars indicate the mean final titer (Log_10_ CCU/mL) of three independent biological replicates (*n* = 3), with error bars representing the standard deviation (SD). Different lowercase letters (a, b, c) above the bars indicate a statistically significant difference between groups (*p* < 0.05); groups not sharing a common letter are significantly different.

Statistical analysis revealed significant differences in growth performance among the media groups for each strain (*p* < 0.05) (Figure 1). For Strain A, the final titers in 20% and 30% HS media were significantly higher than those in all CS media groups. A similar pattern was observed for Strain B, where the 20% HS medium yielded a significantly higher titer compared to all CS groups. For Strain C, titers in the lower concentration media (10% and 20% HS; 10% and 20% CS) were significantly higher than those in the 30% concentration groups. Finally, for Strain D, the 10% and 20% HS groups produced significantly higher titers than all other tested media conditions.

### 3.2. Growth Kinetics Comparison (Time-to-Detection: 10^2^ CCU/mL)

A wide variation was observed in the time required for the different strains to reach the target titer of 10^2^ CCU/mL, with the difference between the fastest and slowest groups spanning up to 16 days. The shortest time was recorded for Strain B in 30% HS medium (3.6 days), while the slowest was observed for Strain D in 20% and 30% CS media (20 days). In Strains A and B, both were generally reached the threshold rapidly, with most conditions (excluding Strain B in 10% HS) taking between 3.6 and 8.6 days. In contrast, Strains C and D were considerably slower, requiring 9 to 20 days. Notably, for every strain, the shortest time-to-detection was consistently achieved in an HS-supplemented medium: Strain A in 20% and 30% HS (4.3 days), Strain B in 30% HS (3.6 days), Strain C in 20% HS (8.3 days), and Strain D in 30% HS (12 days).

The effects of serum type, concentration and strain on growth kinetics were also noted. Within the HS-supplemented groups, the most rapid growth for Strains A, B, and D occurred at the highest concentration (30%), whereas Strain C grew fastest at 20%. The slowest growth in HS was typically observed at the lowest concentration (10% for Strains A, B, D), with the exception of Strain C, which grew slowest at 30%. Among the CS supplemented media, optimal growth kinetics were observed at 20% for Strains A, B, and C, and at 10% for Strain D. Conversely, the longest time-to-detection in CS media was recorded at 30% for Strains A, C, and D, while Strain B exhibited the slowest growth at 10%.

**Figure 2:**
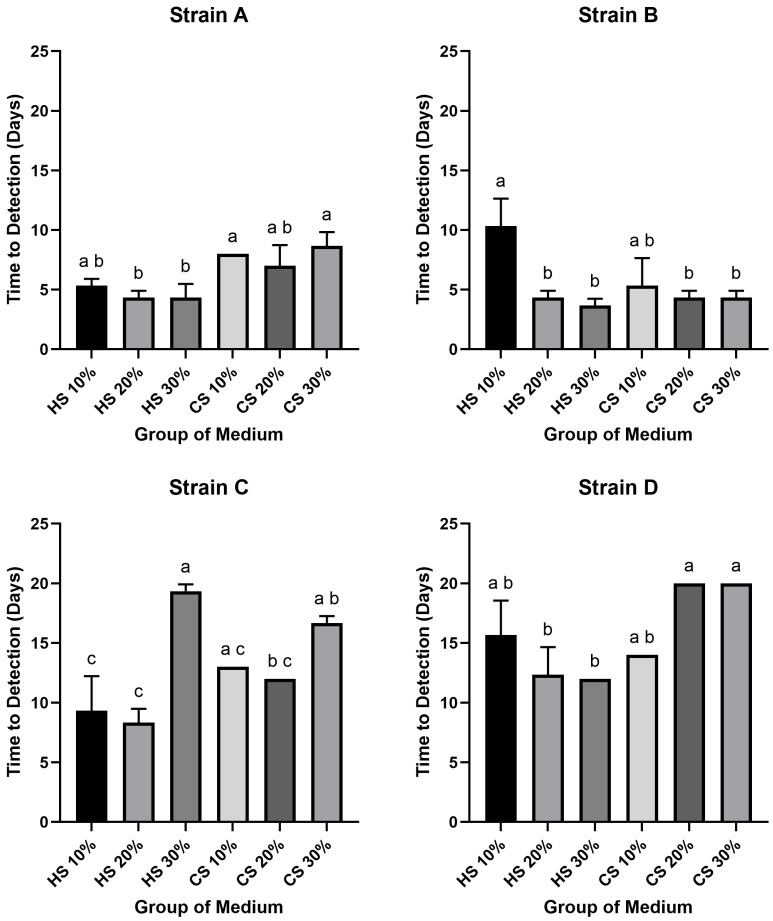
Time-to-detection (10^2^ CCU/mL) for four *Mycoplasma* strains (Strains A–D) cultured in different serum-based media. Each panel illustrates the result for an individual strain: Strains A, B, C and D. The Y-axis represents the time-to-detection in days required to reach a titer of 10^2^ CCU/mL. The X-axis displays the six different medium groups supplemented with various concentrations of HS or CS. Bars indicate the mean values, and error bars represent the standard deviation. Different lowercase letters (a, b, c) above the bars denote a statistically significant difference in the time-to-detection between groups (*p* < 0.05); groups that do not share a common letter are significantly different.

In statistical analysis of the time required to reach the 10^2^ CCU/mL threshold revealed several significant differences (*p* < 0.05) (Figure 2). For Strain A, the time-to-detection for 20% and 30% HS media was significantly shorter than for 10% and 30% CS media. For Strain B, the 10% HS group required a significantly longer period to reach the threshold compared to all other groups, except for the 10% CS group. In the case of Strain C, the 10% and 20% HS groups demonstrated significantly faster growth kinetics than the 30% HS and 30% CS groups; additionally, the 20% CS group was also significantly faster than the 30% HS group. Finally, for Strain D, the 20% and 30% HS media resulted in a significantly shorter time-to-detection compared to the 20% and 30% CS media.

### 3.3. Growth Kinetics Comparison (Time-to-Detection: 10^4^ CCU/mL)

The result of the time required to reach the high-titer threshold of 10^4^ CCU/mL revealed that several groups failed to achieve this concentration. Specifically, Strain C in 30% HS and 30% CS media, as well as Strain D in 30% HS and all CS-supplemented media, did not reach the target titer. Among the successful groups, the most rapid kinetics were observed in HS-supplemented media, with Strain A (in 20% and 30% HS) and Strain B (in 30% HS) all reaching 10^4^ CCU/mL in just 6 days. In contrast, the longest time-to-detection among successful groups was 23 days for Strain D in 10% HS medium. While most successful groups required between 10 and 20 days to reach the target, Strains A and B in several HS media were notable exceptions, demonstrating faster kinetics (6 to 10 days).

Within the HS-supplemented media, the shortest time-to-detection for Strains A and B was at 30%, for Strain C at 10%, and for Strain D at 20%. The longest periods were generally observed at 10% HS for Strains A, B, and D, whereas for Strain C, the 20% HS medium resulted in the slowest growth. In the CS-supplemented media, where only Strains A, B, and C showed any success in reaching the threshold, the most rapid kinetics for Strains A and B were observed at 20% CS. The slowest growth for these strains in CS media occurred at 30% for Strain A and 10% for Strain B. For Strain C, the times-to-detection in 10% and 20% CS were identical.

**Figure 3:**
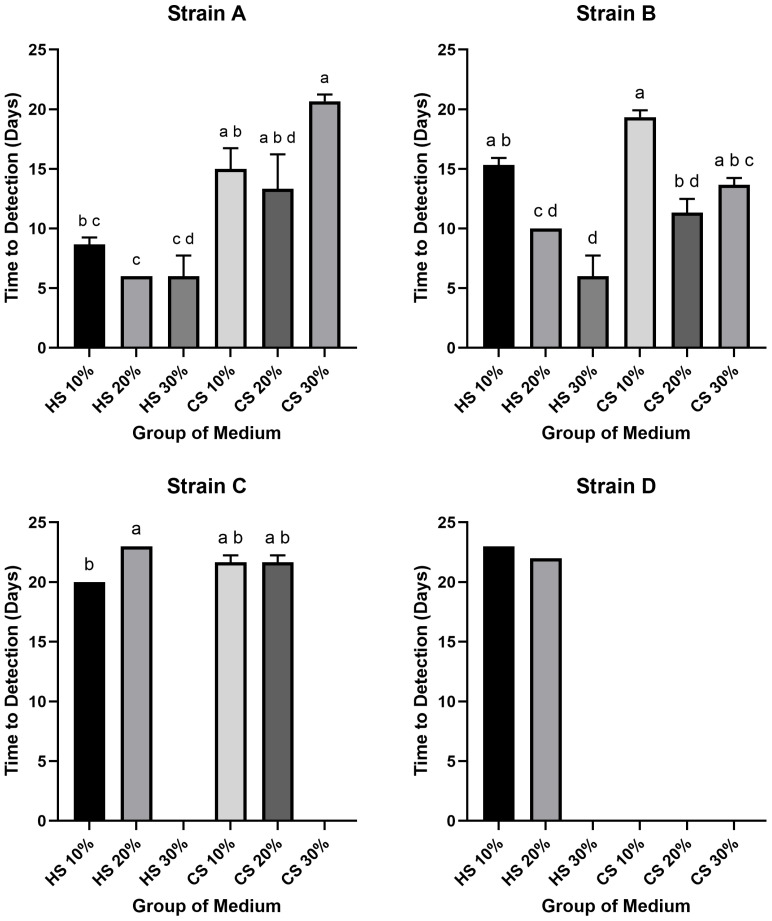
Time-to-detection (10^4^ CCU/mL) for four *Mycoplasma* strains (Strains A–D) cultured in different serum-based media. Each panel illustrates the result for an individual strain: Strains A, B, C and D. The Y-axis represents the time-to-detection in days required to reach the target titer 10^4^ CCU/mL. The X-axis displays the six different medium groups supplemented with various concentrations of HS or CS. Bars indicate the mean values, and error bars represent the standard deviation (*n* = 3). Bars without error bars for Strain D represent the mean of two replicates (*n* = 2) due to one replicate failing to reach the target. The absence of a bar for a specific group indicates that the strain failed to reach the target titer of 10^4^ CCU/mL in that medium within the observation period. Different lowercase letters (a, b, c, d) above the bars denote a statistically significant difference between groups (*p* < 0.05); groups that do not share a common letter are significantly different.

Statistical analysis confirmed significant differences in the time required to reach 10^4^ CCU/mL (*p* < 0.05) (Figure 3). For Strain A, the time-to-detection in 20% HS was significantly shorter than in all CS groups, while 30% HS was significantly shorter than 10% and 30% CS, and 10% HS was significantly shorter than 30% CS. For Strain B, the 30% HS group exhibited significantly faster kinetics than the 10% HS, 10% CS, and 30% CS groups. Furthermore, the 20% HS and 20% CS groups were significantly faster than the 10% HS and 10% CS groups, respectively. For Strain C, the time-to-detection in 10% HS was significantly shorter than in 20% HS. Statistical analysis could not be performed for Strain D due to an insufficient number of groups reaching the target titer.

## 4. Discussion

This study systematically compared horse serum (HS) and chicken serum (CS) for the in vitro cultivation of *M. hyorhinis.* Generally, HS outperformed CS in both final yield and growth kinetics. Regarding final growth yield, HS media enabled strains A and B to reach maximum titers of 10^9^ CCU/mL, a level not achieved under any CS condition tested (Figure 1). A non-linear concentration effect was evident: relative to the 20% HS optimum, 30% HS reduced yields in most strains (*p* < 0.05; Figure 1). This superiority extended to growth kinetics. When assessing the time to reach a 10^2^ CCU/mL threshold, the performance difference was stark: the fastest HS culture required only 3.6 days, whereas the slowest CS culture took 20 days. For the high-titer threshold of 10^4^ CCU/mL, the distinction was even more marked (Figure 2). At the more stringent 10^4^ CCU/mL threshold, several CS groups—particularly for strain D—did not reach the target within the observation window, while the fastest HS groups reaching the target in just 6 days (Figure 3). Collectively, these findings indicate that CS is generally suboptimal for efficient, high-titer propagation, with 20% HS offering a practical balance between speed and yield.

In this study, while both HS and CS were capable of supporting the growth of the tested *M. hyorhinis* strains to varying degrees, significant differences in efficacy were observed. It is well established that *Mycoplasma* species rely on exogenous cholesterol and phospholipids as essential components for membrane synthesis [8,18], and that cholesterol must be efficiently delivered to the membrane via carriers such as high-density lipoprotein (HDL) and albumin [14,19,20]. Previous reports indicate that the total cholesterol content in healthy horse serum typically ranges from 95 to 119 mg/dL, with HDL comprising approximately 26–75% and low-density lipoprotein (LDL) about 15–65% [21,22,23]. In chicken, the serum generally contains higher total cholesterol levels, ranging from approximately 148 to 171 mg/dL depending on breed and husbandry conditions, with both HDL and LDL potentially present at high proportions [24]. These findings suggest that both types of serum possess the potential to support membrane formation in *Mycoplasma* spp. In the current study, however, HS demonstrated a significantly higher final yield and faster growth rate for *M. hyorhinis* compared to CS. The importance of HS for mycoplasmas cultivation has been noted for other species as well. For instance, in a study optimizing the culture medium for *M. gallisepticum*, horse serum was identified as a critical factor for increasing biomass production [13].

Nevertheless, because the cited serum composition data are derived from different species, breeds, ages, and nutritional backgrounds, and do not directly correspond to the specific batches used in this study, the observed superiority of HS over CS cannot be solely attributed to the numerical values of cholesterol and lipoproteins. It is hypothesized that differences in the forms of cholesterol, phospholipid profiles, specific albumin subtypes, or other factors that promote membrane formation and stability may contribute to the enhanced performance of HS. To confirm these hypotheses, future studies employing metabolomic and lipidomic analyses of the specific serum batches used for cultivation are warranted.

The results of this study indicate that CS exhibited limited capacity to support the proliferation of *M. hyorhinis*, which may be attributed to differences in chemical composition and protein structure compared to mammalian serum. Although CS may contain relatively high levels of total cholesterol, HDL, and LDL [25], the form in which cholesterol is present and its binding affinity with lipoproteins and carrier proteins may affect its bioavailability for incorporation into the mycoplasmal membrane. The major carrier proteins such as albumin in CS differ from mammalian albumin in both metal-binding domains and tertiary structure [26]. These structural differences may result in a shift from “specific, high-affinity” metal ion binding to a more “non-specific” mode, potentially causing two adverse outcomes: (1) decreased efficiency in delivering essential metal ions required for mycoplasmal growth, and (2) elevated risk of metal ion toxicity due to the inability to adequately sequester free ions, leading to concentrations that may be harmful to the bacteria. Additionally, previous studies have shown that equine transferrin does not effectively bind to chicken cell transferrin receptors, likely due to the highly species-specific nature of the transferrin–receptor interaction [27]. This finding highlights functional differences between equine and chicken transferrin. Such differences may suggest that the transferrin present in horse and chicken serum differs in its ability to support *Mycoplasma* growth. Chicken transferrin may not efficiently interact with the iron uptake systems of porcine-derived *Mycoplasma* strains, potentially limiting iron availability and consequently restricting bacterial proliferation. Furthermore, immunoglobulin G (IgG) from mammals typically carries complex-type N-glycans at conserved sites, whereas chicken IgG presents a mix of complex-type and high-mannose-type oligosaccharides [28]. A substantial proportion of these are monoglucosylated high-mannose-type structures, which are rarely found in mature mammalian glycoproteins. Such glycan differences may interfere with essential processes such as adhesion or nutrient acquisition, or even exert inhibitory effects, ultimately reducing the proliferation efficiency of *Mycoplasma* in CS-based media. Nonetheless, our study observed that at 20% and 30% serum concentrations, strain B exhibited comparable time to reach 10^2^ CCU/mL in both CS and HS, and only slightly lower growth in CS at 10^4^ CCU/mL. These findings indicate that CS retains a certain degree of supportive potential under specific strain and culture conditions. Given its lower cost and broader availability in some regions, CS warrants further investigation as a cost-effective alternative or component in mixed-serum formulations.

This study observed that HS at a concentration of 20% provided the most favorable growth conditions for *M. hyorhinis*. However, when the concentration was increased to 30%, the final yield of most strains significantly declined, suggesting a concentration-dependent inhibitory effect. Similar results have been reported in other *Mycoplasma* species. For instance, in *M. pneumoniae* cultured in BSF-based medium, increasing serum concentrations from 3% to 6% and 9% enhanced growth, but further elevation to 12% resulted in growth inhibition and reduced overall yield [14]. Washburn and Somerson (1979) also observed a comparable effect, reporting that unfractionated whole serum had a lower growth-promoting effect compared to purified lipoprotein components [20]. These findings led to the hypothesis that whole serum may contain endogenous “toxic or inhibitory substances” that can interfere with bacterial proliferation. In addition, specific serum components may exert direct inhibitory effects. It has been reported that very low-density lipoprotein (VLDL) in human serum can suppress the growth of *M. hominis* when it delivers free cholesterol at concentrations exceeding 10 µg/mL [29]. Taken together, while higher serum concentrations may provide more nutritional resources, they may also lead to the accumulation of inhibitory factors, ultimately impairing bacterial growth.

In this study, although all four strains were isolated from SEP-like lung lesions in field pigs and were preserved and processed under identical conditions, strains A and B consistently exhibited better growth performance across most serum conditions compared to strains C and D. This observation may be attributed to variations among strains in membrane lipid composition, genotypes of nutrient transport systems, or the structure of surface proteins [8]. Such inter-strain differences may underlie the variable growth responses observed under specific culture conditions. Previous studies on *M*. *hyopneumoniae* have demonstrated that significant strain-to-strain variation exists, affecting not only virulence but also the structure of surface lipoproteins involved in nutrient acquisition [30]. This phenomenon is also evident in *M*. *pneumoniae*. For instance, studies have shown that different *M. pneumoniae* strains, including those with varying passage histories and numerous clinical isolates, exhibit inconsistent growth abilities and rates even when cultured in identical media with the same starting concentration [14,16]. Moreover, the response to specific media components can be highly strain-dependent; for instance, one investigation found significant growth variability among *M. pneumoniae* strains, where one prototype strain grew well in a particular egg yolk-based medium while another exhibited poor growth under the same conditions [31]. Interestingly, the growth performance of the strains correlated with their year of isolation in our study; strains A and B (isolated in 2020) consistently outperformed strains C and D (isolated in 2021). This temporal association may reflect genetic or phenotypic shifts that have occurred among field strains over time, potentially influencing their adaptability to different culture environments.

Although this study systematically compared the effects of HS and CS on the in vitro cultivation of multiple *M. hyorhinis* strains, some limitations should be considered. First, the number and origin of strains included in this study were limited and did not encompass all geographic regions or virulence types; thus, the observed results may differ in strains with diverse genetic backgrounds. Second, although a concentration-dependent inhibitory effect of high-concentration HS was observed, its underlying molecular mechanism remains unclear. Further studies incorporating proteomic and lipidomic analyses may be necessary to elucidate the specific components and pathways involved. Third, the initial inoculum titers were not standardized across the four strains. Therefore, direct quantitative comparisons of growth parameters between different strains are not intended and should be interpreted with caution. However, since the primary objective was to compare the performance of the six media for each isolate, the starting titer for all conditions tested within a single strain was identical, thus validating the intra-strain comparisons that form the basis of our conclusions. Finally, this study did not investigate the genomic background of the four isolates. Future studies involving whole-genome sequencing and comparative bioinformatic analysis would be invaluable. Despite these limitations, the findings of this study have implications for diagnostic reagent production and vaccine development. When high-yield cultivation of *M. hyorhinis* is required, 20% HS appears to offer more consistent support. However, in resource-limited settings or when HS supply is unstable, appropriately optimized CS may serve as a feasible alternative. Given that the cost difference between CS and HS is not consistent across brands or regions, practical decisions should be based on local supply conditions and pricing. Furthermore, since even strains of the same species may respond differently to serum components, strain-specific optimization is recommended prior to scaling up to ensure stable yield and product quality.

## 5. Conclusions

This study systematically compared the effects of HS and CS on the in vitro cultivation of *M. hyorhinis*. The results demonstrated that 20% HS significantly outperformed CS in terms of both growth rate and final yield, while avoiding the inhibitory effects observed at higher concentrations (30%). Notable strain-specific differences were observed in response to serum type and concentration, suggesting that variations in strains may influence serum utilization efficiency. Although CS was generally less effective than HS, it still exhibited supportive potential under certain strain and culture conditions, indicating its viability as a low-cost alternative (in some regions) or component in mixed-serum formulations. These findings contribute to the optimization of *Mycoplasma* culture media and have practical implications for the development of diagnostic tools and vaccine manufacturing.

## Figures and Tables

**Table 1:** Comparative summary of growth performance for four *M. hyorhinis* strains in different serum-supplemented media.

Strain	Medium	Final Titer (Log_10_ CCU/mL) (Mean ± SD)	Time to 10^2^ CCU/mL (Days) (Mean ± SD)	Time to 10^4^ CCU/mL (Days) (Mean ± SD)
A	HS 10%	7.0 ± 0	5.3 ± 0.6	8.7 ± 0.6
	HS 20%	9.0 ± 0	4.3 ± 0.6	6.0 ± 0
	HS 30%	9.0 ± 0	4.3 ± 1.2	6.0 ± 1.7
	CS 10%	5.0 ± 0	8.0 ± 0	15 ± 1.7
	CS 20%	5.0 ± 0	7.0 ± 1.7	13.3 ± 2.9
	CS 30%	5.0 ± 0	8.7 ± 1.2	20.6 ± 0.6
B	HS 10%	6.0 ± 0	10.3 ± 2.3	15.3 ± 0.6
	HS 20%	9.0 ± 0	4.3 ± 0.6	10.0 ± 0
	HS 30%	6.0 ± 0	3.6 ± 0.6	6.0 ± 1.7
	CS 10%	4.0 ± 0	5.3 ± 2.3	19.3 ± 0.6
	CS 20%	5.0 ± 0	4.3 ± 0.6	11.3 ± 1.2
	CS 30%	5.0 ± 0	4.3 ± 0.6	13.7 ± 0.6
C	HS 10%	4.3 ± 0.6	9.3 ± 2.9	20 ± 0
	HS 20%	4.0 ± 0	8.3 ± 1.2	23 ± 0
	HS 30%	2.0 ± 0	19.3 ± 0.6	N/A ^1^
	CS 10%	4.0 ± 0	13.0 ± 0	21.7 ± 0.6
	CS 20%	4.0 ± 0	12.0 ± 0	21.7 ± 0.6
	CS 30%	2.0 ± 0	16.7 ± 0.6	N/A ^1^
D	HS 10%	3.7 ± 0.6	15.7 ± 3.1	23.0 (*n* = 2) ^2^
	HS 20%	3.7 ± 0.6	12.3 ± 2.3	22.0 (*n* = 2) ^2^
	HS 30%	2.0 ± 0	12.0 ± 0	N/A ^1^
	CS 10%	2.0 ± 0	14.0 ± 0	N/A ^1^
	CS 20%	2.0 ± 0	20.0 ± 0	N/A ^1^
	CS 30%	2.0 ± 0	20.0 ± 0	N/A ^1^

^1^ N/A: Not Achieved within the 30-day observation period. ^2^ For this condition, one of the three replicates did not reach the target and was excluded from the analysis.

## Data Availability

The data presented in this study are available in the article.

## Abbreviations

The following abbreviations are used in this manuscript:

BSF	Bovine Serum Fraction
CCU	Color Changing Unit
CS	Chicken Serum
HDL	High-Density Lipoprotein
HS	Horse Serum
IgG	Immunoglobulin G
LDL	Low-Density Lipoprotein
qPCR	Quantitative Polymerase Chain Reaction
SD	Standard Deviation
SEP	Swine Enzootic Pneumonia
VLDL	Very Low-Density Lipoprotein
